# Whole planarian chromosome squash

**DOI:** 10.1016/j.xpro.2020.100257

**Published:** 2021-01-09

**Authors:** Paul G. Barghouth, Néstor J. Oviedo

**Affiliations:** 1 Department of Molecular & Cell Biology, University of California, Merced, Merced, CA, USA; 2 Quantitative and Systems Biology Graduate Program, University of California, Merced, Merced, CA, USA; 3 Health Sciences Research Institute, University of California, Merced, Merced, CA, USA

**Keywords:** Model organisms, Molecular biology

## Abstract

Whole planarian chromosome squash allows researchers to qualitatively analyze chromosome integrity. Treatment with colchicine is used to halt dividing cells within metaphase and does not require amputation or tissue puncturing. In combination with acetic-orcein, a stain-fixative for chromosomes, this strategy is suitable for animals with friable tissues caused by drug treatment, radiation, and RNA interference phenotypes. The whole planarian squash method presented here is a minimally invasive procedure that facilitates simultaneous analysis of chromosomal integrity in control and experimental animals.

For complete details on the use and execution of this protocol, please refer to Peiris et al. (2016).

## Highlights

• Analysis of whole planarian chromosome integrity
• Minimally invasive and does not require procedures such as amputation or puncture
• Feasible in friable tissues resulting from pharmacological treatments or RNAi phenotypes
• Analysis in parallel of control and experimental samples

## Graphical Abstract

**Figure:**
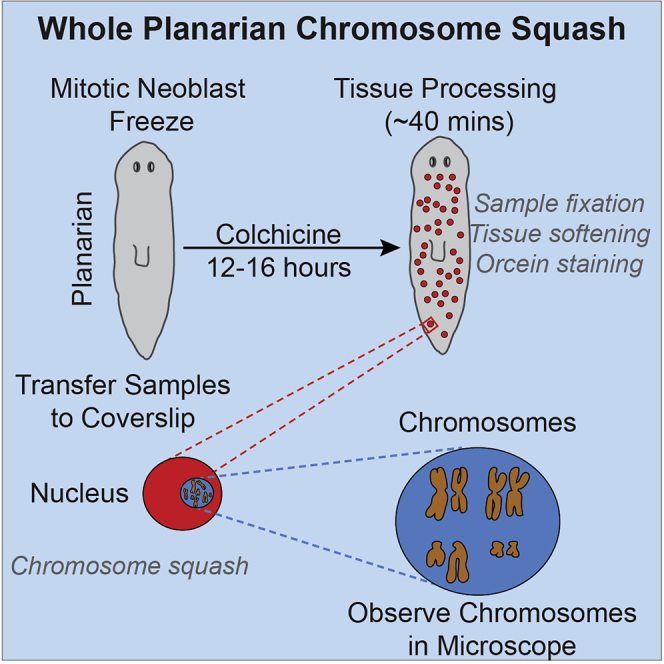

## Before you begin

On day 1 of the protocol fresh colchicine solution should be made. Prior to the start of day 2 of the protocol, all karyotyping reagents must be made fresh.

### Colchicine solution

**Timing: 5 min**

1. Prepare fresh 0.05% colchicine solution.
  a. Weight 0.005 g of 97% colchicine.
  b. Dilute in 10 mL of planarian 1× Montjuic saltwater.
  c. Vortex until powder dissolves.

### Karyotyping reagents

**Timing: 30–60 min**

2. Perform tissue fixation in 3:1 ethanol: acetic acid.
3. Prepare 1 N HCl by diluting 12.1 N HCl in MilliQ water.
4. Prepare 60% acetic acid solution in MilliQ water.
5. Prepare 1:1:1 ratio of lactic acid, acetic acid, and MilliQ water.
6. Preparation of 1% acetic-orcein solution
  a. Weigh 1 g orcein.
  b. Dissolve in 45 mL of hot (e.g., near boiling) acetic acid.
  c. Once dissolved, allow solution to cool.
  d. Add 55 mL of MilliQ water, shake well, and filter.

## Key resources table

**Table:** 

REAGENT or RESOURCE	SOURCE	IDENTIFIER
**Chemicals, peptides, and recombinant proteins**		

97% colchicine	ACROS	Cat# 227120010
Orcein	Sigma-Aldrich	Cat# O7380
Lactic acid	Fisher Scientific	Cat# A159-500
Glacial acetic acid	Fisher Scientific	Cat# A38-500
Hydrochloric acid 12.1 N	Fisher Scientific	Cat# A144-500
Ethyl alcohol denatured	Fisher Scientific	Cat# A407-4

**Experimental models: organisms/strains**		

Planarian: *Schmidtea mediterranea*	n/a	*CIW4*

**Software and algorithms**		

ImageJ	https://imagej.nih.gov	Version 1.48
NIS Elements AR	Nikon	Version 3.2

**Other**		

100× objective	Nikon	n/a
Nikon AZ-100 multi-zoom	Nikon	n/a
24 × 60 mm cover slips	Fisher Scientific	Cat# 12-545-89
22 × 22 mm cover slip	Fisher Scientific	Cat# 12547
6 cm petri dish	Fisher Scientific	Cas# FB0875713A
Thermomixer-R or Precision GP	Eppendorf Thermo Scientific	Cat# EP022670107 Cat# TSGP02
1.5 mL centrifuge tubes	Fisher Scientific	Cat# 05-408-129
15 mL conical tubes	Fisher Scientific	Cat# 07-200-886
Transfer pipettes (2 mL)	Fisher Scientific	Cat# 13-711-42
Orbital platform rotator	n/a	n/a
Montjuic saltwater or Instant ocean sea salt	Instant Ocean	n/a

## Step-by-step method details

**Figure 1:**
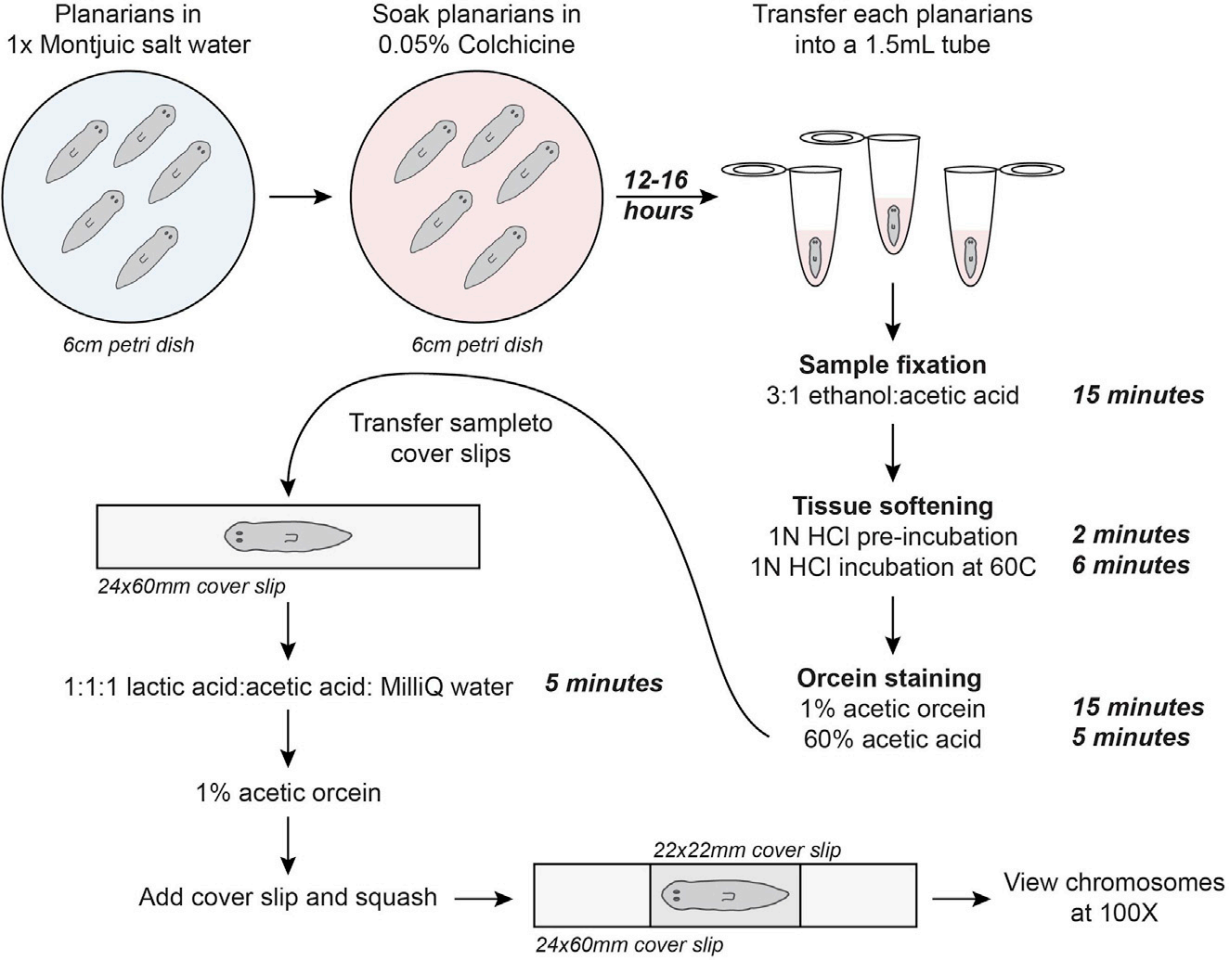
Workflow of whole planarian chromosome squash protocol Step-by-step breakdown and visualization of the whole planarian chromosome squash protocol.

Figure 1 provides an overview of the protocol.

### Colchicine overnight incubation

**Timing: 12–16 h**

Soaking planarians into colchicine solution to freeze mitotic neoblasts.

1. Select control and/or experimental planarians of similar size that are no greater than 1 cm in length and place them into a 6 cm petri dish.
2. Remove planarian 1× Montjuic saltwater and replace with 4–5 mL of Colchicine solution.
3. Allow planarians to incubate for 12–16 h in the dark.

**CRITICAL:** To avoid planarian toxicity induced by colchicine, the incubation period should not exceed 16 h.

### Whole planarian chromosome squash

**Timing: 30–60 min**

**Figure 2:**
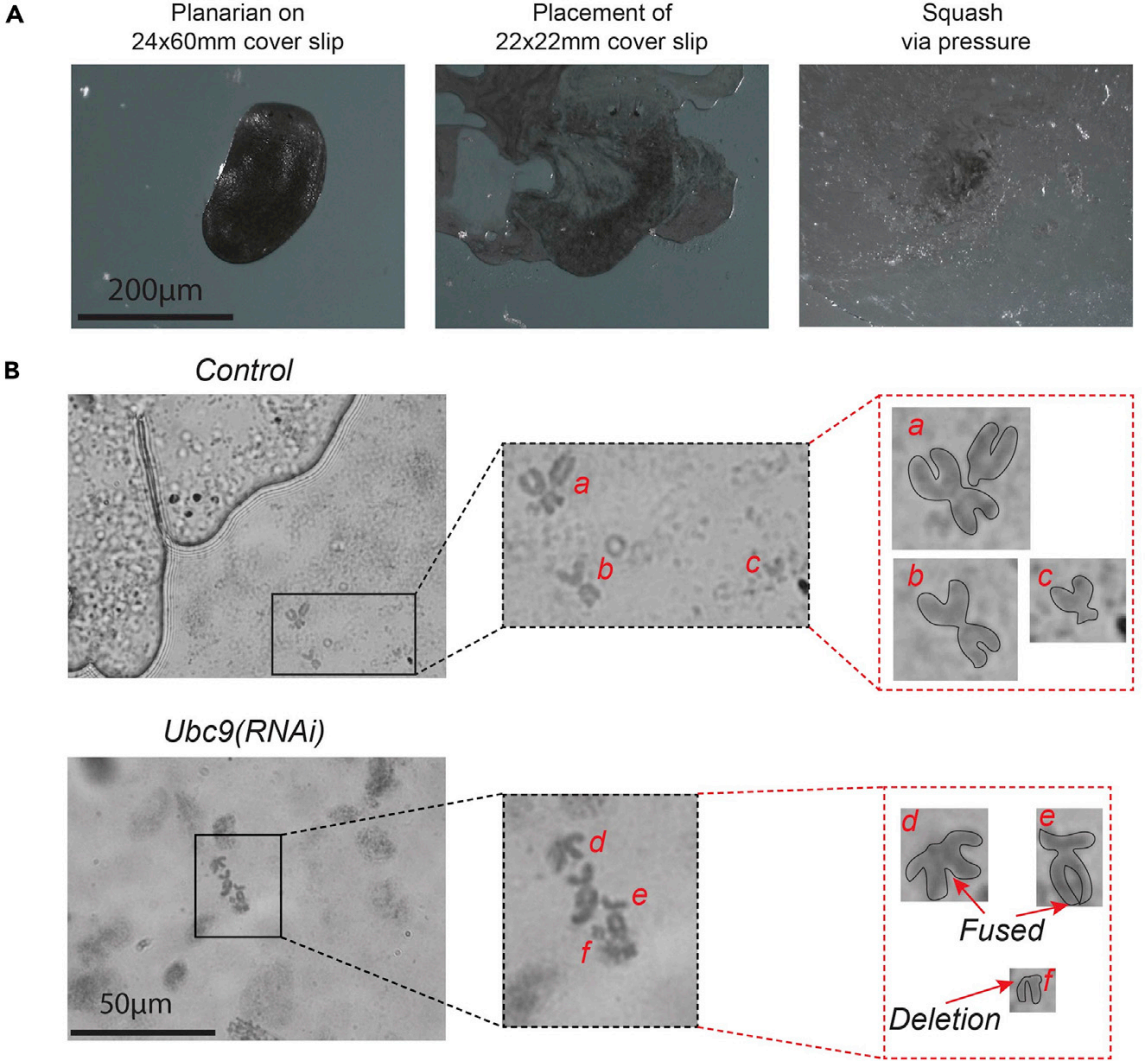
Whole planarian chromosome squash and chromosome visualization (A) Representative images of planarian squash. Left image is a planarian on a 24 × 60 mm coverslip. Middle image is of the addition of a 22 × 22 mm coverslip on top of the planarian, the initial squashing. Right image is of a fully squashed planarian after application of pressure to the added coverslip. Scale bar, 200 μm. (B) Representative images and zoom-ins of chromosome obtained from squashed control and *Ubc9(RNAi)* animals. *Ubc9* is required for DNA damage repair within the planarian, thus, without its function chromosomal abnormalities, such as fusions and deletions, are evident (d, e, f) relative to the control (a, b, c). Scale bar, 50 μm.

Figure 2A provides a visual representation of the whole animal procedure.

Planarian tissue will be fixed and heated, assisting with the softening of tissue and separation of cells allowing for chromosomes to be stained by orcein solution.

4. Place one animal per 1.5 mL tube and remove colchicine solution.
5. Add 0.5–1 mL of 3:1 ethanol: acetic acid fixative to each tube and incubate for 15 min at room temperature (25°C) on an orbital platform rotator.

***Note:*** In all steps, ensure planarians are fully submerged within the solution and not floating on top.

6. Replace solution with 0.5–1 mL 1 N HCl and pre-incubate for 2 min at room temperature (25°C) .
7. Immediately incubate at 60°C in a Thermomixer-R for 6 min.
8. Quickly remove the HCl solution.

**CRITICAL:** At this point tissues are quite soft. Thus, remove all residual HCl without puncturing the planarian by angling the 1.5 mL tube so that the planarian will stick to the wall of the tube.

9. Add 150–200 μL 1% acetic-orcein solution to each tube and incubate at room temperature (25°C) for 15 min.
10. Carefully remove the 1% acetic-orcein solution.

**CRITICAL:** The planarian will not be seen, as the 1% acetic-orcein solution is dark purple. In most cases, the planarian will be residing at the bottom of the tube. Thus, remove as much solution as possible (∼125–175 μL). The residual liquid will be diluted out with 60% acetic acid solution.

11. Add 500 μL of 60% acetic acid solution and incubate for 5 min.
12. With a transfer pipette gently place animals on a 24 × 60 mm cover slips.

***Note:*** 25 × 75 mm slides are too thick to view chromosomes at 100× thus, use 24 × 60 mm cover slips.

13. After planarians are transferred, remove residual 60% acetic acid solution.
14. Add 10–20 μL of 1:1:1 lactic acid: acetic acid: MilliQ H_2_O on top of each worm and incubate for 5 min.
15. Remove excess liquid and add 2 μL of orcein solution to each animal.
16. Take a 22 × 22 mm cover slip, quickly place it on top of the treated animal and with slight pressure and one continual movement, use your thumb to squash the animal throughout the slide. Troubleshooting 1.

**Pause point:** Protocol is complete. Slides can be stored at room temperature (25°C) until chromosome viewing. Slides do not require sealing and can be stored at room temperature (25°C) for long-term void of humidity.

### Viewing planarian chromosomes

**Timing: 1–2 h**

Figure 2B provides representative illustrations of normal and abnormal chromosomes.

Visualization of planarian chromosomes using a 100× objective.

17. View chromosomes with a 100× objective. Troubleshooting 1. Troubleshooting 2.
18. Take 20–30 representative fields per animal.

## Expected outcomes

Whole planarian chromosome squash is designed to obtain the maximum number of chromosomes per animal without manipulating tissue (e.g., amputations or tissue puncturing) prior to the colchicine soaking step. Phenotypes of weakened tissue integrity and lesions are commonly found within experimental groups (e.g., *Ubc9(RNAi)* and *Rad51(RNAi)* animals) (Peiris et al., 2016; Thiruvalluvan et al., 2018). Thus, reduced tissue manipulation allows for the analysis of chromosomes between control and experimental groups.

The asexual strain of the planarian species *Schmidtea mediterranea* consist of four diploid chromosomes (Guo et al., 2018; Knakievicz et al., 2007; Newmark and Alvarado, 2002). Upon manipulation of key genes involved in planarian DNA damage repair, chromosomal abnormalities arise, such as dicentric, telomeric fusions, acentric fragments and deletions (Barghouth et al., 2019; Peiris et al., 2016; Thiruvalluvan et al., 2018).

## Limitations

This protocol is limited to brightfield imaging as the animal is squashed onto a slide with a coverslip. Other protocols have been optimized to allow for immunohistochemical staining and telomeric FISH protocols within planarian (Guo et al., 2018). Future work is required to optimize whole planarian chromosome squashing for immunohistochemical staining and FISH protocols with minimal disruption to squashed tissue.

## Troubleshooting

There are two possible issues that may arise during this protocol. The issues will not be evident until viewing under a microscope and will result in the inability to view chromosomes. The potential issues are (1) excessive squashing of sample and (2) suboptimal orcein staining.

### Problem 1: excessive squashing

If too much pressure is used during the squashing process, chromosomes will be damaged, destroyed or squashed to the point that they cannot be viewed under the microscope (step 16).

### Potential solution

The amount of force required to conduct planarian squash must be optimized per scientist. If thumb pressure is excessive, try using an unsharpened pencil and drop the pencil eraser down onto the planarian for squashing.

### Problem 2: suboptimal orcein staining

Depending on the species of planarian, 1% acetic-orcein may not be efficient for chromosome staining. Therefore, a limited number or no chromosomes will be visible (steps 9 and 14).

### Potential solution

The percent of orcein must be optimized per species. Studies have shown that increasing orcein concentration to 2% could enhance visualization of chromosomes (Cour, 2009).

### Resource availability

#### Lead contact

Further information and requests for resources and reagents should be directed to and will be fulfilled by the Lead Contact, Néstor J. Oviedo (noviedo2@ucmerced.edu).

#### Materials availability

This study did not generate new unique reagents

#### Data and code availability

This study did not generate/analyze datasets/code
